# The PAF1 complex cell autonomously promotes oogenesis in *Caenorhabditis elegans*

**DOI:** 10.1111/gtc.12938

**Published:** 2022-04-27

**Authors:** Yukihiro Kubota, Natsumi Ota, Hisashi Takatsuka, Takuma Unno, Shuichi Onami, Asako Sugimoto, Masahiro Ito

**Affiliations:** ^1^ Department of Bioinformatics College of Life Sciences, Ritsumeikan University Kusatsu Japan; ^2^ Advanced Life Sciences Program Graduate School of Life Sciences, Ritsumeikan University Kusatsu Japan; ^3^ RIKEN Center for Biosystems Dynamics Research Kobe Japan; ^4^ Laboratory of Developmental Dinamics Graduate School of Life Sciences, Tohoku University Sendai Japan

**Keywords:** *C. elegans*, germ cell, germ line development, OMA‐1, oocytes, oogenesis, PAFO‐1::mCherry transgene, RNA polymerase II‐associated factor 1 complex, RNAi, transcriptional regulation

## Abstract

The RNA polymerase II‐associated factor 1 complex (PAF1C) is a protein complex that consists of LEO1, RTF1, PAF1, CDC73, and CTR9, and has been shown to be involved in RNA polymerase II‐mediated transcriptional and chromatin regulation. Although it has been shown to regulate a variety of biological processes, the precise role of the PAF1C during germ line development has not been clarified. In this study, we found that reduction in the function of the PAF1C components, LEO‐1, RTFO‐1, PAFO‐1, CDC‐73, and CTR‐9, in *Caenorhabditis elegans* affects oogenesis. Defects in oogenesis were also confirmed using an oocyte maturation marker, OMA‐1::GFP. While four to five OMA‐1::GFP‐positive oocytes were observed in wild‐type animals, their numbers were significantly decreased in *pafo‐1* mutant and *leo‐1(RNAi)*, *pafo‐1(RNAi)*, and *cdc‐73(RNAi)* animals. Expression of a functional PAFO‐1::mCherry transgene in the germline significantly rescued the oogenesis‐defective phenotype of the *pafo‐1* mutants, suggesting that expression of the PAF1C in germ cells is required for oogenesis. Notably, overexpression of OMA‐1::GFP partially rescued the oogenesis defect in the *pafo‐1* mutants. Based on our findings, we propose that the PAF1C promotes oogenesis in a cell‐autonomous manner by positively regulating the expression of genes involved in oocyte maturation.

## 1 INTRODUCTION

During animal development, spatiotemporal regulation of gene transcription is essential for precise regulation of cell behavior. To precisely regulate gene transcription, the recruitment and activation of RNA polymerase II (Pol II) to the transcriptional target is required. In addition, chromatin remodeling affects DNA accessibility during transcription through epigenetic modification of nucleosomes. The RNA polymerase II‐associated factor 1 (PAF1) complex, or PAF1C, is a highly conserved protein complex in eukaryotes, which is involved in multiple aspects of Pol II‐mediated transcriptional regulation, including transcriptional elongation, 3′‐end processing, and epigenetic modification. Moreover, the PAF1C is involved in the post‐transcriptional step of gene expression and translational regulation via its interaction with the regulatory sequences of mRNAs (Francette et al., 2021; Jaehning, 2010).

The PAF1C was originally identified in *Saccharomyces cerevisiae* as an RNA pol II interactor (Shi et al., 1996, 1997; Wade et al., 1996). It consists of five subunits (Leo1, Rtf1, Paf1/pancreatic differentiation, Cdc73/parafibromin, and Ctr9) (Mueller et al., 2004; Mueller & Jaehning, 2002). Although PAF1C is not essential for the viability of *S. cerevisiae*, depletion or mutation of the PAF1 subunits causes severe developmental disorders during the development of somite, neural crest, neuron, heart, and craniofacial cartilage in zebrafish (Akanuma et al., 2007; Jurynec et al., 2019; Langenbacher et al., 2011; Nguyen et al., 2010). Additionally, the PAF1C affects Notch, Wnt, and Hedgehog signaling (Akanuma et al., 2007; Mosimann et al., 2006, 2009; Tenney et al., 2006). The PAF1C has also been reported to regulate the proliferation, differentiation, morphology, cell migration, epidermal morphogenesis, mitophagy, maintenance of stem cells, and tumorigenesis (Bai et al., 2010; Carpten et al., 2002; Ding et al., 2009; Kubota et al., 2014; Langenbacher et al., 2011; Lin et al., 2008; Moniaux et al., 2006; Ponnusamy et al., 2009; Shi et al., 1996; Zhang et al., 2013; Zheng et al., 2020). However, the functional importance of the PAF1C in germ cell development has not yet been explored.

The development of the nematode, *Caenorhabditis elegans*, is highly reproducible, which makes it a reliable model organism for analyzing the regulatory mechanism of development. The hermaphrodite gonad of this nematode temporally produces sperm at the late larval stage, which are stored in the spermatheca, and subsequently produces oocytes during the adult stage. During this process, spatiotemporal regulation of gene expression, cell proliferation, cell differentiation, cell shape change, cell growth, and meiotic progression occurs (Arur, 2017; Huelgas‐Morales & Greenstein, 2018; Kim et al., 2013). However, the mechanism of oogenesis has not been fully elucidated.

In this study, we found that all the PAF1C components are involved in promoting oogenesis, and that the expression of OMA‐1, a CCCH‐type zinc finger protein involved in oocyte maturation, is promoted by the PAF1C in a cell‐autonomous manner.

## 2 RESULTS

### 2.1 The PAF1C is essential for oogenesis

**FIGURE 1:**
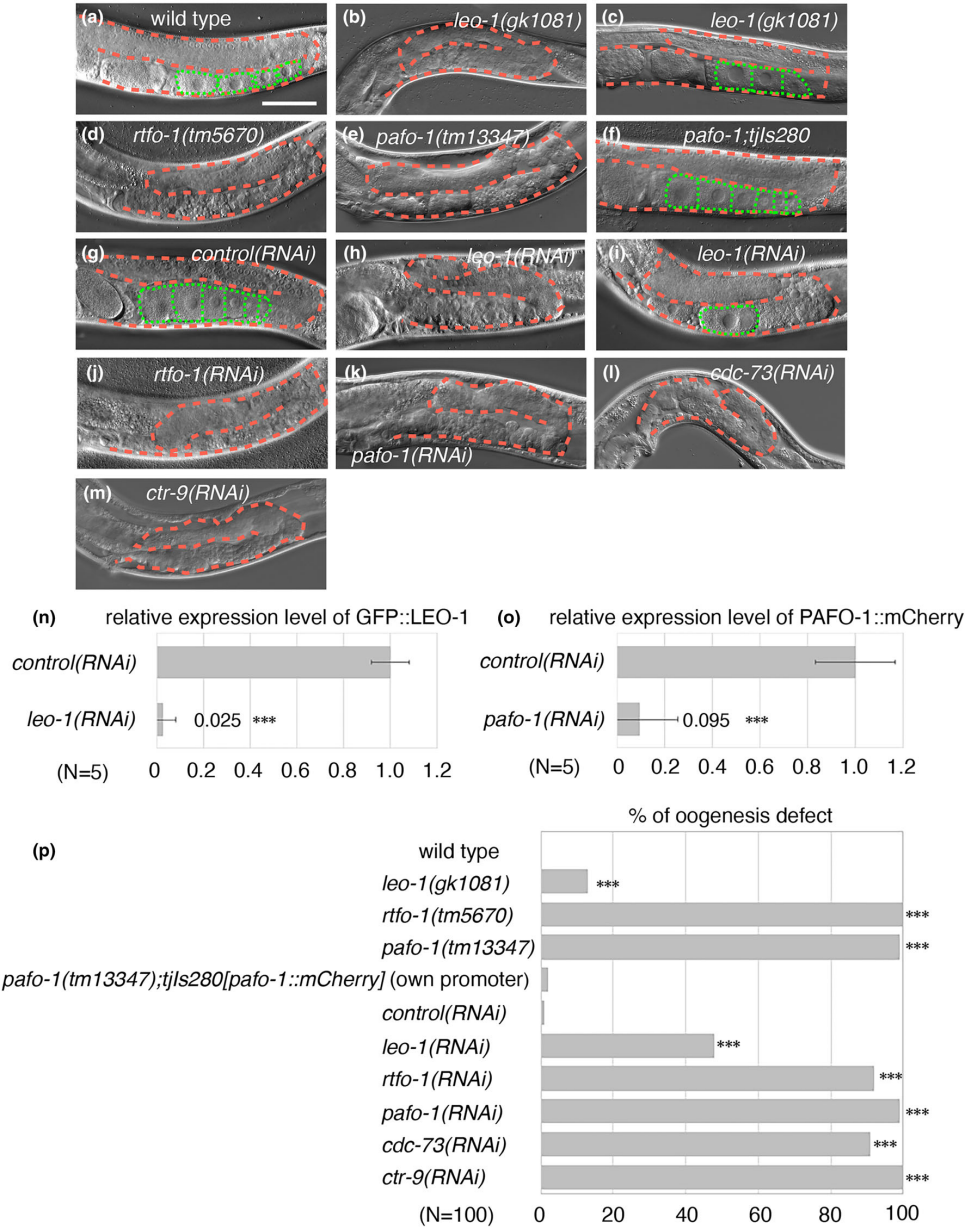
PAF1C is essential for oogenesis. (a–m) Germ cell development of wild‐type (a), *leo‐1(gk1081)* (b, c), *rtfo‐1(tm5670)* (d), *pafo‐1(tm13347)* (e), *pafo‐1(tm13347);tjIs280[pafo‐1p::pafo‐1::mCherry::pafo‐1 3′‐UTR]* (f), *control(RNAi)* (g), *leo‐1(RNAi)* (h, i), *rtfo‐1(RNAi)* (j), *pafo‐1(RNAi)* (k), *cdc‐73(RNAi)* (l), and *ctr‐9(RNAi)* (m) in hermaphrodite day 1 adult posterior gonads. (n, o) Quantitative analysis of the RNAi efficiency of *leo‐1(RNAi)* (n) and *pafo‐1(RNAi)* (o). *P*‐values are indicated for Student's *t*‐test in comparison with the *control(RNAi)*. ***p < .005. The error bars represent ±SD. See Figures S1 and S2 for more information about the fluorescent images. (p) Percentages of oogenesis defects found in 1 day‐adult wild type, mutants, transgenic rescued, *control(RNAi)*, and RNAi‐knockdown animals of each PAF1C component. *P*‐values are indicated for Fisher's exact test in comparison with WT or *control(RNAi)*. ***p < .005. Error bars represent ±SD. In all the panels, the anterior region of the gonad was to the left, and the dorsal region was at the top of the image. The posterior gonads are shown. The orange dotted lines mark the gonad boundaries and the green dotted lines mark the oocyte boundaries. Scale bar (white), 50 μm

To analyze whether the PAF1C is involved in germ cell development in *C. elegans*, we observed germ cell development of the posterior gonads in day 1 adults of the PAF1C mutants by DIC microscopy (Figure 1). Compared with the wild‐type animals, oogenesis was insufficient in the PAF1C mutants, *leo‐1(gk1081)*, *rtfo‐1(tm5670)*, and *pafo‐1(tm13347)* mutants, although the penetrance of *leo‐1(gk1081)* was lower than that of the other mutants (Figure 1a–1e and 1p). An integrated transgene, *tjIs280[pafo‐1::mCherry]*, expressed by the *pafo‐1* regulatory region (Figure 4a,h), rescued the oogenesis defect of the *pafo‐1(tm13347)* deletion mutant (Figure 1f,p). Similar to the observations of deletion mutants, although the penetrance of the oogenesis defect of the *leo‐1(RNAi)* was relatively low, all the five RNAi‐knockdown animals of the PAF1C components exhibited oogenesis defects (Figure 1g–m,p). Next, we analyzed the expression patterns of LEO‐1 and PAFO‐1 by using animals expressing the *tjIs308[leo‐1p::GFP::leo‐1]* and *tjIs280[pafo‐1p::pafo‐1::mCherry]* transgenes by authentic promoters. The results indicated that both of the GFP::LEO‐1 and PAFO‐1::mCherry were ubiquitously expressed within the germ cells (Figures S1 and S2). The efficiency of both *leo‐1(RNAi)* and *pafo‐1(RNAi)* was over 90% (Figure 1n,o; Figures S1 and S2), as measured by the fluorescent signal of GFP::LEO‐1 and PAFO‐1::mCherry, respectively, at the distal region of the gonad. These results suggest that the PAF1C is essential for oogenesis, and that the contribution of LEO‐1 to the PAF1C function may be the lowest among the PAF1C components.

### 2.2 The PAF1C is dispensable for spermatogenesis

**FIGURE 2:**
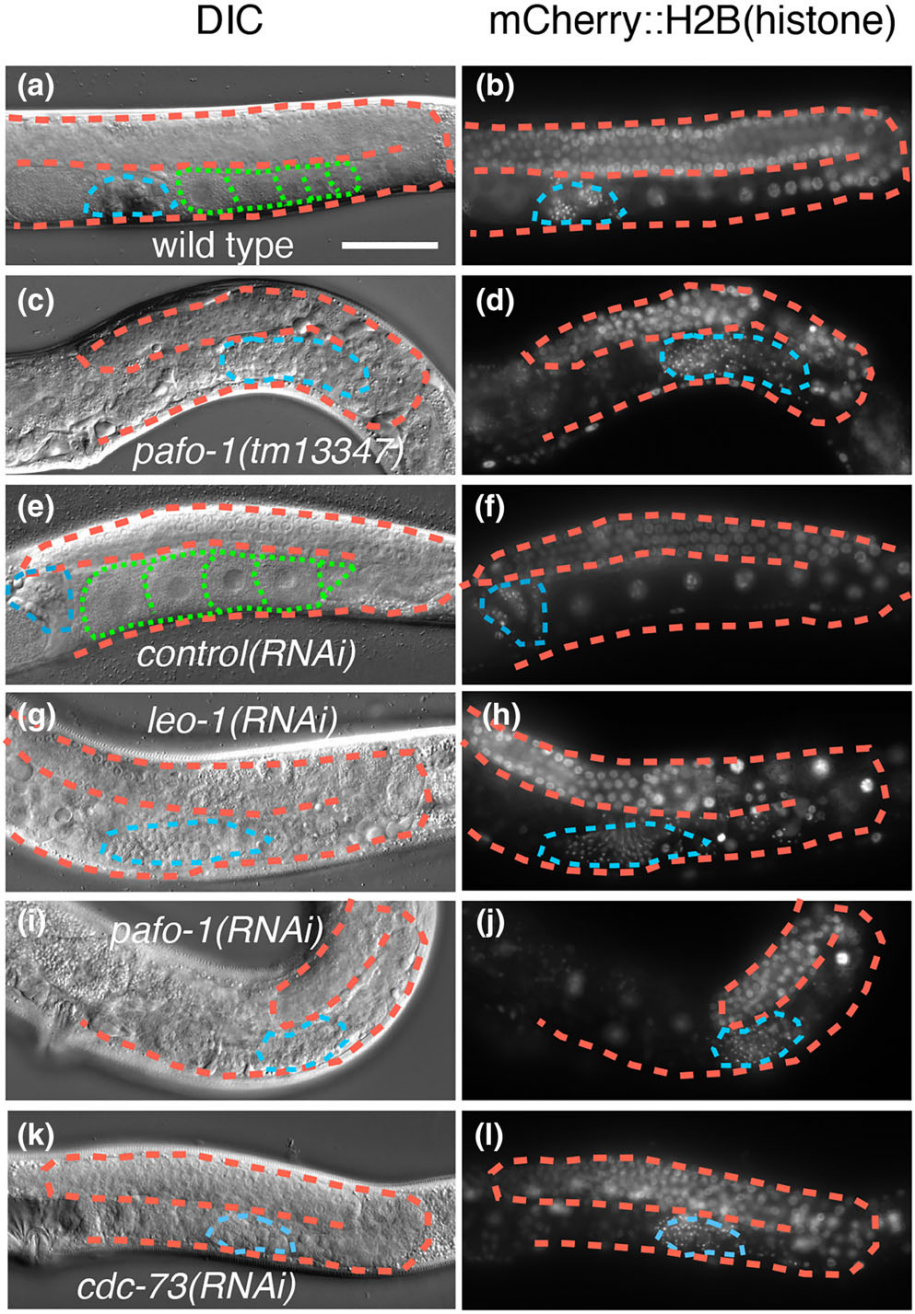
PAF1C is dispensable for spermatogenesis. (a–l) Differential interference contrast (DIC) (a, c, e, g, i, and k) and fluorescence (b, d, f, h, j, and l) images of wild type (a, b), *pafo‐1(tm13347)* (c, d), *control(RNAi)* (e, f), *leo‐1(RNAi)* (g, h), *pafo‐1(RNAi)* (i, j), and *cdc‐73(RNAi)* (k, l) day 1 adult animals with *tjIs57[pie‐1p::mCherry::H2B::pie‐1 3′‐UTR]*. In all the panels, the anterior region of the gonad is to the left, and the dorsal region is at the top of the image. The posterior gonads are shown. The orange dotted lines mark the gonad boundaries, the green dotted lines mark the oocyte boundaries and the blue dotted lines surround the sperm‐like small cells. Scale bar (white), 50 μm

Next, we examined whether the PAF1C is involved in spermatogenesis. When nuclei were visualized with mCherry::H2B(histone), sperm‐like small cells were detected in wild type and *control(RNAi)* animals. Similarly, sperm‐like cells were formed in *pafo‐1(tm13347)*, *leo‐1(RNAi)*, *pafo‐1(RNAi)*, and *cdc‐73(RNAi)* animals (Figure 2). Thus, these results suggest that the PAF1C is not essential for spermatogenesis.

### 2.3 The PAF1C is involved in the expression of OMA‐1 in oocytes

**FIGURE 3:**
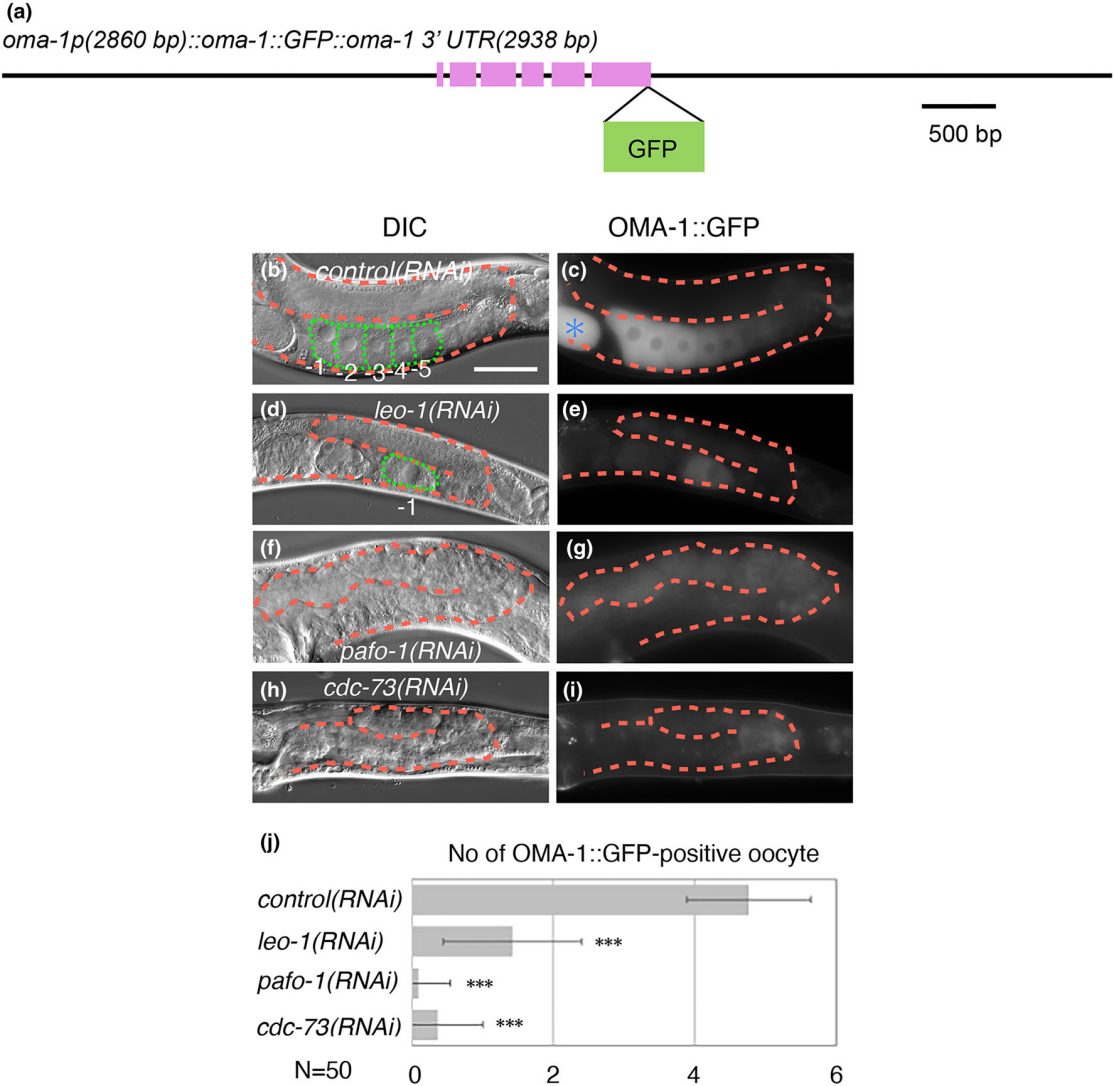
PAF1C is essential for the promotion of oocyte maturation. (a) Genomic structure of the translational GFP‐fusion construct of *oma‐1*. (b–i) Differential interference contrast (DIC) (b, d, f, and h) and fluorescence (c, e, g, and i) images of *control(RNAi)* (b, c), *leo‐1(RNAi)* (d, e), *pafo‐1(RNAi)* (f, g), and *cdc‐73(RNAi)* (h, i) day 1 adult animals with *bkcSi11[oma‐1p::oma‐1::GFP::oma‐1 3′‐UTR]* (c, e, g, and i). (j) Quantification of OMA‐1::GFP‐positive oocytes. *P*‐values are indicated for Student's *t*‐test in comparison with *control(RNAi)*. ***p < .005. The error bars represent ±SD. In all the panels, the anterior region of the gonad is to the left, and the dorsal region is at the top of the image. Posterior gonads are shown. The orange dotted lines mark the gonad boundaries and the green dotted lines mark the oocyte boundaries. Asterisk (blue) indicates fertilized egg. Scale bar (white), 50 μm

Next, we investigated how the PAF1C regulates oogenesis. To visualize matured oocytes, we used an oocyte maturation marker, OMA‐1::GFP, which was derived from the *oma‐1* regulatory region (Figure 3a). In the day 1 adult stage of *control(RNAi)* animals, 4.8 OMA‐1::GFP‐positive cells were arranged linearly in the ventral region of each gonad on an average (*N* = 50, Figure 3b,c,j). In contrast, the average number of OMA‐1::GFP‐positive cells were 1.4, 0.10, and 0.36 in *leo‐1(RNAi)*, *pafo‐1(RNAi)*, and *cdc‐73(RNAi)* animals, respectively (*N* = 50, Figure 3d–j). These results suggest that the PAF1C positively regulates the expression of OMA‐1.

### 2.4 Germ cell‐specific expression of PAFO‐1::mCherry Rescues the oocyte maturation‐defective phenotype of the *pafo‐1(tm13347)* mutant

**FIGURE 4:**
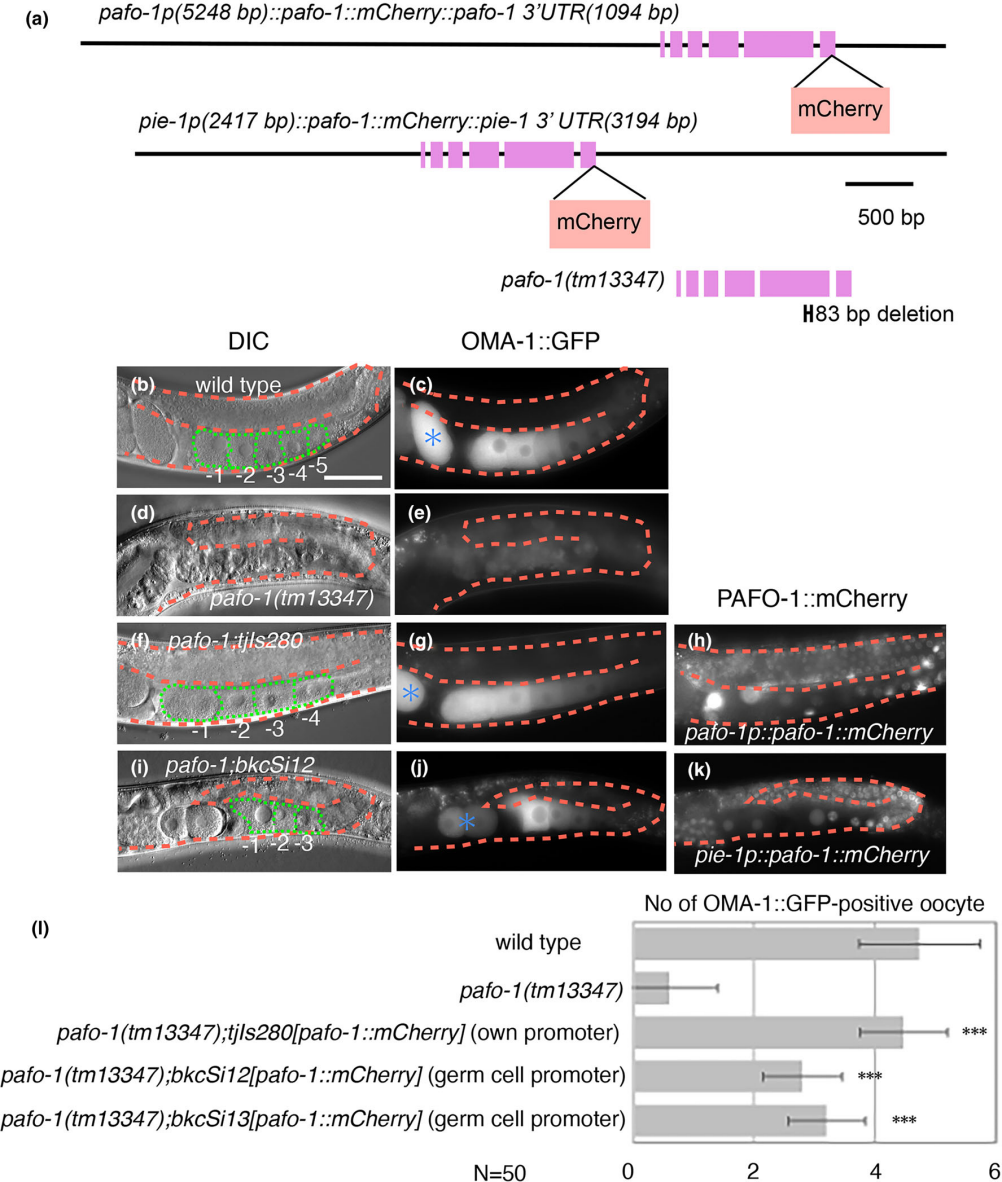
PAF1C promotes oocyte maturation in a cell‐autonomous manner. (a) Genomic structures of the translational mCherry‐fusion construct of *pafo‐1*, germ cell specific mCherry‐fusion construct of *pafo‐1*, and the deleted region of *pafo‐1(tm13347)*. (b–k) Differential interference contrast (DIC) (b, d, f, and i) and fluorescence (c, e, g, h, j, and k) images on wild type (b, c), *pafo‐1(tm13347)* (d, e), *pafo‐1(tm13347);tjIs280[pafo‐1p::pafo‐1::mCherry::pafo‐1 3′‐UTR]* (f–h), *pafo‐1(tm13347);bkcSi12[pie‐1p::pafo‐1::mCherry::pie‐1 3′‐UTR]* (i–k) of day 1 adult animals with *bkcSi11[oma‐1p::oma‐1::GFP::oma‐1 3′‐UTR]*. c, e, g, and j indicate the OMA‐1::GFP signals from the GFP channel. h and k indicate the *tjIs280*‐derived PAFO‐1::mCherry signals from the mCherry channel, and the *bkcSi12*‐derived PAFO‐1‐mCherry signals from the mCherry channel, respectively. (l) Quantification of OMA‐1::GFP‐positive oocytes. *P*‐values are indicated for Student's *t*‐test in comparison with *pafo‐1(tm13347)*. ***p < .005. The error bars represent ±SD. In all the panels, the anterior region of the gonad is to the left, and the dorsal region is at the top of the image. Posterior gonads are shown. The orange dotted lines mark the gonad boundaries and the green dotted lines mark the oocyte boundaries. Asterisk (blue) indicates fertilized egg. Scale bar (white), 50 μm

To determine the tissue in which the expression of PAF1C is required for oocyte maturation, we performed a tissue‐specific rescue experiment using the *pafo‐1(tm13347)* deletion mutant. In the day 1 adult stage of wild‐type animals, approximately 4.7 OMA‐1::GFP‐positive cells were arranged linearly in the ventral region of each gonad on an average (*N* = 50, Figure 4b,c,l). In contrast, the number of OMA‐1::GFP‐positive cells was significantly decreased in the *pafo‐1(tm13347)* deletion mutant (the average number of OMA‐1::GFP‐positive cells was 0.6, *N* = 50, Figure 4d,e,l). Introduction of the *tjIs280[pafo‐1::mCherry]* transgene, expressed by the *pafo‐1* regulatory region, almost completely rescued the oogenesis defect (the average number of OMA‐1::GFP‐positive cells was 4.5, *N* = 50, Figure 4f–h,l). Similarly, when we introduced integrated *pafo‐1::mCherry* transgenes by germ cell‐specific regulatory regions of *pie‐1*, *bkcSi12* (Figure 4a,k), and *bkcSi13* (Figure 4a), they significantly rescued the oogenesis defect of the *pafo‐1(tm13347)* deletion mutant (the average number of OMA‐1::GFP‐positive cells from *pafo‐1(tm13347);bkcSi12;bkcSi11* and *pafo‐1(tm13347);bkcSi13;bkcSi11* was 2.8 and 3.2, respectively, *N* = 50, Figure 4i–l). These results suggest that the PAF1C regulates oogenesis in a cell‐autonomous manner. Although the rescue activity with regard to the number of OMA‐1::GFP‐positive cells was not complete, *bkcSi13[pie‐1p::pafo‐1::mCherry::pie‐1 3′‐UTR]* rescued the sterility of the *pafo‐1(tm13347)* mutant, and the *pafo‐1(tm13347);bkcSi13[pie‐1p::pafo‐1::mCherry::pie‐1 3′‐UTR]* survived and produced the next generation both in the presence and absence of *bkcSi11[oma‐1p::oma‐1::GFP::oma‐1 3′‐UTR]*. Thus, germ cell expression of PAFO‐1::mCherry is sufficient for the formation of functional oocytes and its maternal contribution is sufficient for embryonic, larval, larval–adult transition, and germ cell development in the next generation.

### 2.5 Overexpression of OMA‐1::GFP Partially rescues the oogenesis defect in the *pafo‐1(tm13347)* mutant

**FIGURE 5:**
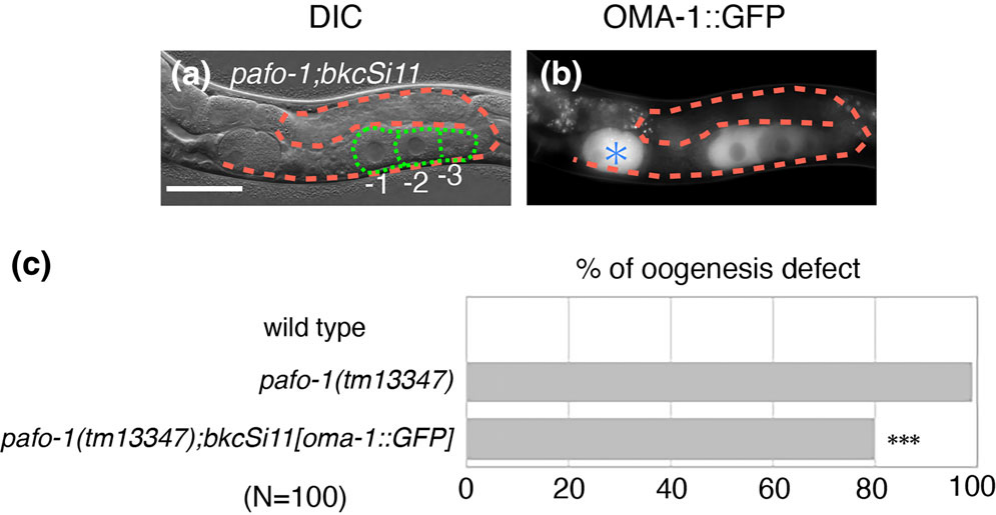
Overexpression of OMA‐1::GFP partially rescues the oogenesis defect in the *pafo‐1(tm13347)* mutant. (a, b) Differential interference contrast (DIC) (a) and fluorescence (b) images of *pafo‐1(tm13347);bkcSi11[oma‐1p::oma‐1::GFP::oma‐1 3′‐UTR]* day 1 adult animal. (c) Percentages of oogenesis defects found for 1 day‐adult wild type, *pafo‐1(tm13347)* mutant, and *pafo‐1(tm13347);bkcSi11[oma‐1p::oma‐1::GFP::oma‐1 3′‐UTR]* animals. *P*‐value is indicated for Fisher's exact test in comparison with *pafo‐1(tm13347)*. ***p < .005. In all the panels, the anterior region of the gonad is to the left, and the dorsal region is at the top of the image. Posterior gonad is shown. The orange dotted lines mark the gonad boundaries and the green dotted lines mark the oocyte boundaries. Asterisk (blue) indicates fertilized egg. Scale bar (white), 50 μm

We tested whether the reduction in the expression of OMA‐1 is the major cause of oogenesis defects in the PAF1C mutants. When OMA‐1::GFP was overexpressed in the *pafo‐1(tm13347)* mutant, the oogenesis defect was partially rescued (Figure 5). Therefore, a possible role of the PAF1C in the germline is to promote oogenesis by positively regulating the expression of *oma‐1*.

## 3 DISCUSSION

The PAF1C is a highly conserved protein complex that consists of five conserved components, namely LEO1, RTF1, PAF1, CDC73, and CTR9. Although it has been shown to be required in diverse biological processes, its contribution to germ cell development has not yet been explored. In this study, we performed functional analysis of the PAF1C in the germ cell development of *C. elegans* and demonstrated its requirement for the promotion of oogenesis and expression of OMA‐1 during oogenesis.

Although PAFO‐1 is ubiquitously expressed in germ cells, OMA‐1 is expressed exclusively in maturing oocytes (Detwiler et al., 2001). This distribution implies that PAFO‐1 could act as a regulator of OMA‐1 expression during the oocyte maturation process. Consistent with this hypothesis, the OMA‐1 expression level was found to be significantly decreased in *pafo‐1* mutants, suggesting that the PAF1C may function as a positive regulator of OMA‐1 expression.

Although the PAF1C components, LEO‐1 and PAFO‐1, are expressed ubiquitously, including in germ cells (Kubota et al., 2014), the PAF1C is required only for oogenesis but not for spermatogenesis. Because the PAF1C is not required for sperm formation, it is unlikely that the oogenesis‐defective phenotype is causative of earlier defects in germ cell development. These results suggest that PAF1C regulates a specific set of genes that are required for oogenesis.

**FIGURE 6:**
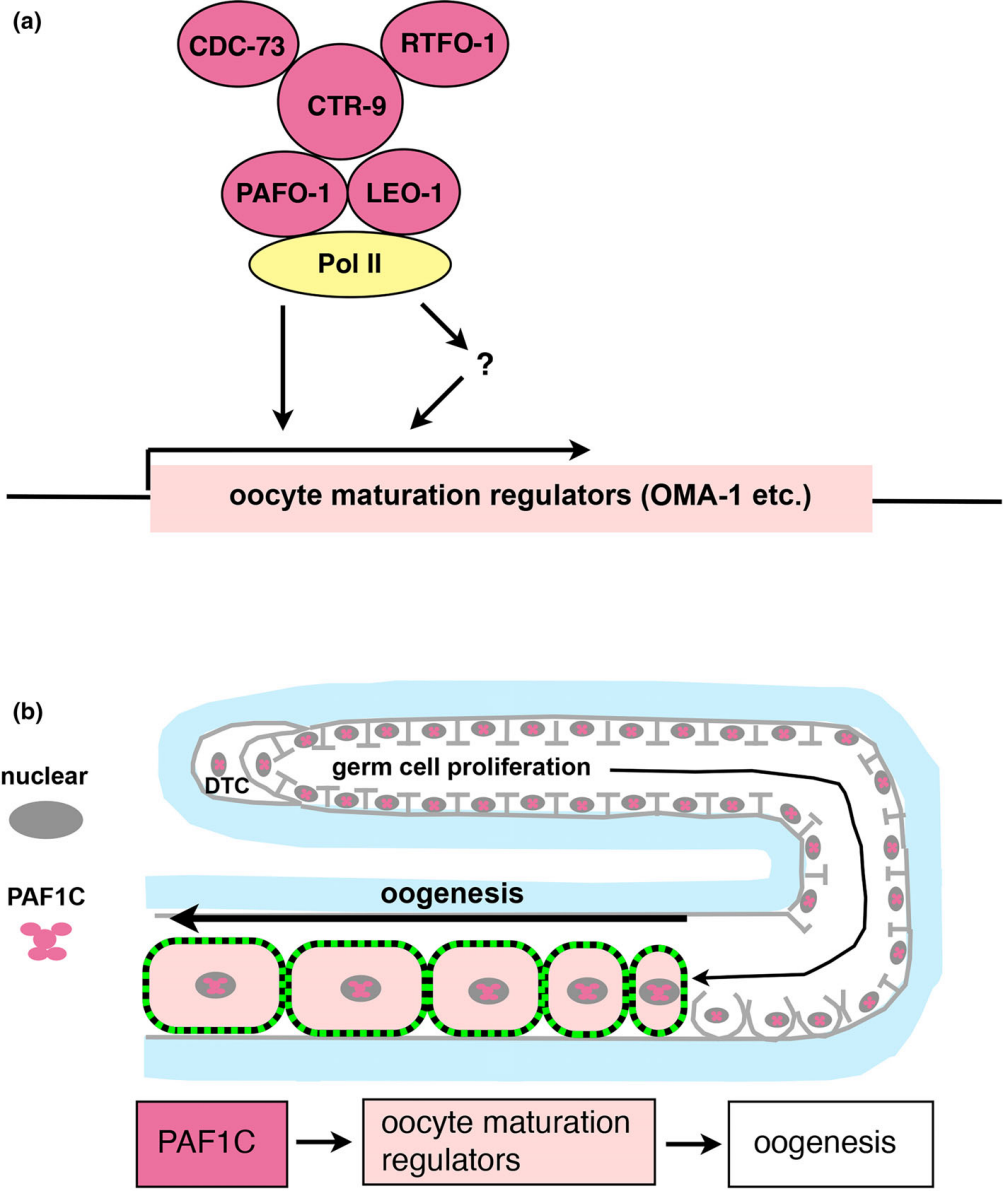
Models for the PAF1C‐dependent regulation of oogenesis. (a) The PAF1C and RNA polymerase II (RNA pol II) are recruited to the regulatory region of target genes, and directly or indirectly promote the expression of the oocyte maturation regulators. (b) During oocyte maturation, the PAF1C promotes oocyte maturation regulators, which then promotes oogenesis in the ventral region of the gonad

The number of OMA‐1::GFP‐positive maturing oocytes was decreased in *leo‐1(RNAi)*, *pafo‐1(RNAi)*, and *cdc‐73(RNAi)* animals and in *pafo‐1* deletion mutants. Therefore, one possible role of the PAF1C is the promotion of OMA‐1 expression. We also show that the overexpression of OMA‐1::GFP partially rescued the oogenesis defect in the *pafo‐1* mutant. Therefore, the PAF1C may promote oogenesis by positively regulating the specific set of downstream oocyte maturation regulators, including OMA‐1 (Figure 6). Our data also suggest that the germ cell expression of PAF1C is sufficient for the formation of fully functional oocytes and that the maternal contribution of the PAF1C is sufficient for embryonic development, larval development, and larval–adult transition.

It has been shown that *oma‐1* and *oma‐2* act redundantly to promote the later part of oocyte maturation to complete oocyte maturation (Detwiler et al., 2001; Spike et al., 2014; Tsukamoto et al., 2017). In contrast, the expansion defect of oocytes in the PAF1C‐depleted animals and mutants occurred in the early part of the oocyte maturation process. Although the overexpression of OMA‐1::GFP partially rescued the oogenesis defect of the *pafo‐1* mutant, the phenotypic similarity of the oogenesis defect was not observed in the *pafo‐1(tm13347)* mutant and the *oma‐1(RNAi);oma‐2(RNAi)* animals. These results indicate that the phenotypic severity of PAF1C‐depleted animals was stronger than that of OMA‐1/2‐double depleted animals. Therefore, it is pertinent to discuss as to why the overexpression of OMA‐1 rescues the failure of oocyte expansion phenotype of the *pafo‐1* mutant. A possible explanation is that although the major function of PAF1C is to promote the expression of OMA‐1/2, the PAF1C may also regulate other targets that are involved in the promotion of the expansion process of oocytes in parallel with the OMA‐1/2 functions. Further analysis is required to confirm this hypothesis.

In this study, we found that oogenesis defects were less severe in *leo‐1(RNAi)* animals among the animals with RNAi‐knockdown of the five components of the PAF1C. Similar to our observations, RTF1, PAF1, CDC73, CTR9, but not LEO1, were reported to be required for the specification of an appropriate number of cardiomyocytes and for elongation of the heart tube in zebrafish (Langenbacher et al., 2011). Taken together, these observations suggest that in the specific context of the differentiation process, among the five PAF1C components, the requirement for LEO‐1 is less critical, and this difference is conserved in vertebrates and invertebrates. At present, there are several possible avenues for exploring this phenomenon. In yeast, Ctr9, Cdc73, and Rtf1, but not Leo1, were shown to require Paf1 at normal levels, and loss of Cdc73 resulted in a lower abundance of Rtf1 (Mueller et al., 2004; Squazzo et al., 2002). Therefore, it is expected that the other four components may achieve only a part of the PAF1C function in the absence of LEO‐1.

There are several possible mechanisms to explain why the *leo‐1(gk1081)* mutant exhibited less severe defects in germ cell development. Recently, crystal structure analysis revealed that the reconstituted PAF1C quaternary Ctr9/Paf1/Cdc73/RTf1 complex may act as the core module of the yeast PAF1C in the absence of the Leo1 subunit (Chen et al., 2022). The mutant LEO‐1(*gk1081*) protein was shown to have lost more than two‐thirds of the carboxyl‐terminal region (Kubota et al., 2014), which include the PAF1‐binding domain of the *Homo sapiens* LEO1 protein (Chu et al., 2013). Therefore, the mutant LEO‐1(*gk1081*) protein might be excluded from the LEO‐1‐containing complex. Because Leo1 appears to mediate the interaction between the PAF1C and elongating Pol II through its RNA–binding activity in yeast (Dermody & Buratowski, 2010), in parallel to the LEO‐1‐dependent function of the PAF1C, an unknown LEO‐1‐independent PAF1C function may also be involved in the PAF1C‐mediated regulation of the oogenesis in *C. elegans*. Therefore, it is conceivable that the *leo‐1(gk1081)* mutant achieves the LEO‐1‐independent regulation of *C. elegans* oogenesis in consequence of physical interaction among the components of the quaternary complex.

Although all the PAF1C components are required for its function, each component has a specific role in regulating the expression of gene encoding cell differentiation regulators, possibly by affecting the formation of the protein complex, specific protein–protein interactions, and protein–DNA/RNA interactions. Further studies are required to determine how PAF1C regulates tissue‐specific development in multicellular organisms.

## 4 EXPERIMENTAL PROCEDURES

### 4.1 *Caenorhabditis elegans* strains

*Caenorhabditis elegans* strains used in this study were derived from the wild‐type (WT) Bristol strain N2 (Brenner, 1974). Worms were incubated at 20°C, except those that were fed RNAi bacteria and were maintained at 22°C.

The *leo‐1* locus encodes a predicted polypeptide of 430 amino acids (aa), and the *gk1081* allele (isolated by the *C. elegans* Gene Knockout Consortium) deleted 627 bp that would result in a C‐terminally truncated protein of 137 aa (intrinsic 132 aa with an extra 5 aa) (Kubota et al., 2014). The *rtfo‐1* locus encodes a predicted polypeptide of 613 aa, and the *tm5670* allele (isolated by the National Bioresource Project Japan) deleted 361 bp that would result in a C‐terminally truncated protein product of 349 aa (intrinsic 314 aa with an extra 35 aa). The *pafo‐1* locus encodes a predicted polypeptide of 425 aa, and the *tm13347* allele (isolated by the National Bioresource Project, Japan) deleted 83 bp that would result in a C‐terminally truncated protein product of 288 aa (intrinsic 285 aa with an extra 3 aa). The C‐terminus of Rtf1 in yeast has been shown to be required for its efficient anchoring to the PAF1C; *rtfo‐1(tm5670)* is expected to be a complex formation‐defective mutant (Warner et al., 2007). As mentioned above, *leo‐1(gk1081)* is not a protein‐null mutant. The *leo‐1(gk1081)* mutant has been shown to produce reduced amounts of C‐terminally truncated proteins in a previous study (Kubota et al., 2014). At present, we have not checked whether or not *rtfo‐1(tm5670)* and *pafo‐1(tm13347)* are null mutants.

We also used the following alleles for construction of mutants: *tjIs57[pie‐1p::mCherry::H2B::pie‐1 3*′*‐UTR + unc‐119(+)]* (Toya et al., 2011), *bkcSi11[oma‐1p::oma‐1::GFP::oma‐1 3*′*‐UTR*, *NeoR] IV*, *bkcSi12[pie‐1p::pafo‐1::mCherry::pie‐1 3*′*‐UTR*, *NeoR]*, *bkcSi13[pie‐1p::pafo‐1::mCherry::pie‐1 3′‐UTR*, *NeoR]* (this work), *tjIs280[pafo‐1p::pafo‐1::mCherry::pafo‐1 3′‐UTR*, *Cbr‐unc119(+)]*, *tjIs308[leo‐1p::GFP::leo‐1::leo‐1 3′‐UTR*, *Cbr‐unc‐119(+)]* (Kubota et al., 2014), *TmC3V[TmIs1230]*, and *TmC5 IV[tmIs1220]* (Dejima et al., 2018).

To obtain *leo‐1(gk1081)* homozygote hermaphrodites, *leo‐1(gk1081)* was balanced with *TmC5 IV[tmIs1220]*, and Venus‐negative homozygote progeny was scored. To obtain *rtfo‐1(tm5670) and pafo‐1(tm13347)* homozygote hermaphrodites, *rtfo‐1(tm5670)* and *pafo‐1(tm13347)* were balanced with *TmC3V[TmIs1230]*, and mCherry‐negative homozygote progeny was scored.

The strains used in this work are listed in Table S1.

### 4.2 Plasmid construction

The plasmids used in this study are listed in Table S2. A miniMos backbone vector, denoted as pYK13, was constructed by inserting a 376 bp fragment containing a multicloning site into a StuI site in pCFJ910. To construct the targeting vectors, the following fragments were amplified and individually subcloned into pYK13 at the NotI and AscI sites. To construct transgenes that expressed GFP‐fusion proteins from putative endogenous 5′‐cis regulatory regions of *oma‐1*, the genomic fragments, *oma‐1::GFP* derived from the *oma‐1* regulatory region (2860 bp), the coding region, and the *oma‐1* 3′‐UTR (2935 bp), were PCR amplified and then fused with GFP.

For germ cell‐specific expression experiments, the *pafo‐1* genome was subcloned into a carboxyl‐terminal mCherry‐fusion protein expression vector, which has cis regulatory regions of *pie‐1*, pYK229, a modified vector derived from pYK13.

### 4.3 Strain construction for rescue experiments

Transgenic worms were prepared by microinjection of the target gene (Mello et al., 1991). Strains that expressed *oma‐1::GFP* under putative endogenous 5′‐cis regulatory regions and 3′‐cis regulatory region of *oma‐1*, and strains that expressed PAFO‐1::mCherry under the germ cell‐specific regulatory regions of *pie‐1* were used for miniMos methods (see below). Single‐copy transgenic‐insertion worms were generated using the miniMos method (Frokjaer‐Jensen et al., 2014) for genomic GFP/mCherry‐fusion expression and tissue‐specific rescue experiments with the wild‐type as the host strain. For microinjections, the following mixtures were used: 10 μg/ml each of GFP/mCherry‐tagged miniMos‐target transgene (*oma‐1p::oma‐1::GFP::oma‐1 3′‐UTR + NeoR* plasmid pYK29, *pie‐1p::pafo‐1::mCherry:: pie‐1 3′‐UTR + NeoR* plasmid pYK232); transposase pCFJ601, 50 μg/ml; injection markers *Prab‐3::mCherry::unc‐54 3′‐UTR* plasmid pGH8, 10 μg/mL; *Pmyo‐2::mCherry::unc‐54 3′‐UTR* plasmid pCFJ90, 2.5 μg/ml; *Pmyo‐3::mCherry::unc‐54 3′‐UTR* plasmid pCFJ104, 5 μg/ml; and pBluescript II KS(−), 30 μg/ml; negative selection marker *Phsp‐16.41::peel‐1::tbb‐2 3′‐UTR* plasmid pMA122, 10 μg/ml.

The integrated alleles, *tjIs280[pafo‐1p::pafo‐1::mCherry::pafo‐1 3′‐UTR*, *Cbr‐unc119(+)]*, *bkcSi12[pie‐1p::pafo‐1::mCherry::pie‐1 3′‐UTR*, *NeoR]*, and *bkcSi13[pie‐1p::pafo‐1::mCherry::pie‐1 3′‐UTR*, *NeoR]* were introduced to the *pafo‐1(tm13347)* mutant or *bkcSi11 [oma‐1p::oma‐1::GFP::oma‐1 3′‐UTR*, *NeoR]*; *pafo‐1(tm13347)* mutant background. Day 1 adult worms were used to score the oogenesis defects.

### 4.4 Feeding RNAi

The worms were fed on RNAi‐feeding plates as previously described (Kamath et al., 2001). Full‐length *leo‐1*, *rtfo‐1*, *pafo‐1*, and *cdc‐73* cDNAs and 1000 bp *ctr‐9* (1st–1000th coding region) cDNA were isolated from a *C. elegans* cDNA library and inserted into the feeding RNAi vector, L4440. An L4440 vector lacking an insert was used as a *control(RNAi)*. After confirming that each inserted sequence was correct, the feeding vectors were individually transformed into *Escherichia coli* HT115 (DE3) samples, which were then seeded separately onto plates of nematode growth medium agar containing Luria–Bertani medium and 50 μg/ml ampicillin, and incubated overnight at 37°C. Thereafter, each culture was seeded onto a 60 mm feeding agar plate containing 50 μg/ml ampicillin and 1 mM isopropyl β‐d‐1‐thiogalactopyranoside and incubated at 23°C for 2 days. L4‐stage worms were transferred to a feeding plate and cultured at 22°C. Phenotypes of F1 worms were determined at the day 1 adult stage.

### 4.5 Microscopy

Fluorescence and differential interference contrast; DIC microscopy procedures were performed using an Olympus BX63 microscope with an ORCA‐Spark camera (Hamamatsu Photonics) and UPlanSApo X60 water NA 1.20 or UPlanXApo X40 NA 0.95 objective lens (Olympus). The microscope system was controlled using the cellSens Dimension software (Olympus). Images were processed using the ImageJ (NIH) or Adobe Photoshop 2021 software.

### 4.6 Analyses of the efficiency of RNAi knockdown of *leo‐1* and *pafo‐1*

Fluorescence signals of GFP::LEO‐1 and PAFO‐1::mCherry from images of the RNAi‐treated animals at the perinuclear region of the germ cells of the distal gonad obtained under the fluorescence/DIC microscope, fluoresce signals were measured using Image J software (National Institutes of Health; see Figure 1n,o). A rectangular area (8 μm × 40 μm) was used to analyze the GFP/mCherry signals. The relative expression levels of GFP::LEO‐1 or PAFO‐1::mCherry were then calculated and compared between *control(RNAi)* and *leo‐1(RNAi)* or *pafo‐1(RNAi)* animals, respectively.

### 4.7 Statistical analyses

The *P‐*values from the Fisher's exact test for the percentage of animals with oogenesis defects in *leo‐1(gk1081)*, *rtfo‐1(tm5670)*, and *pafo‐1(tm13347)* were calculated for comparison with wild‐type animals. The *p‐*values from the Fisher's exact test for the percentage of animals with oogenesis defects in *leo‐1(RNAi)*, *rtfo‐1(RNAi)*, *pafo‐1(RNAi)*, *cdc‐73(RNAi)*, and *ctr‐9(RNAi)* were calculated for comparison with control *(RNAi)* animals. For the OMA‐1::GFP overexpression experiment, the *p‐*values from the Fisher's exact test for the percentage of animals with oogenesis defects in *pafo‐1(tm13347);bkcSi11[oma‐1p::oma‐1::GFP::oma‐1 3′‐UTR*, *NeoR]* was calculated for comparison with *pafo‐1(tm13347)* animals.

The *p‐*value for the Student's *t*‐test of the relative expression level of GFP::LEO‐1 at the distal gonad‐arm region in *leo‐1(RNAi);tjIs308[leo‐1p::GFP::leo‐1::leo‐1 3′‐UTR*, *Cbr‐unc‐119(+)]* was calculated for comparison with the *control(RNAi);tjIs308[leo‐1p::GFP::leo‐1::leo‐1 3′‐UTR*, *Cbr‐unc‐119(+)]* animals. The *p‐*value for the Student's *t*‐test of the relative expression level of PAFO‐1::mCherry at the distal gonad‐arm region in *pafo‐1(RNAi);tjIs280[pafo‐1p::pafo‐1::mCherry::pafo‐1 3′‐UTR*, *Cbr‐unc119(+)]* was calculated for comparison with the *control(RNAi); tjIs280[pafo‐1p::pafo‐1::mCherry::pafo‐1 3′‐UTR*, *Cbr‐unc119(+)]* animals. For rescue experiments, the *p‐*value for the Student's *t*‐test of the number of OMA‐1::GFP positive oocytes in *pafo‐1(tm13347);tjIs280[pafo‐1p::pafo‐1::mCherry::pafo‐1 3′‐UTR];bkcSi11[oma‐1p::oma‐1::GFP::oma‐1 3′‐UTR*, *NeoR]*, *pafo‐1(tm13347);bkcSi12[pie‐1p::pafo‐1::mCherry::pie‐1 3′‐UTR];bkcSi11[oma‐1p::oma‐1::GFP::oma‐1 3′‐UTR*, *NeoR]*, *pafo‐1(tm13347);bkcSi13[pie‐1p::pafo‐1::mCherry::pie‐1 3′‐UTR*]; *bkcSi11[oma‐1p::oma‐1::GFP::oma‐1 3′‐UTR*, *NeoR]* were calculated for comparison with the *pafo‐1(tm13347);bkcSi11[oma‐1p::oma‐1::GFP::oma‐1 3′‐UTR*, *NeoR]* animals.

## Supporting information


**Table S1** *Caenorhabditis elegans* strains constructed for this study.
**Table S2** Plasmids constructed for this study.

**FIGURE S1** Analysis of the efficiency of RNAi knockdown of *leo‐1*.
(a–l) Differential interference contrast (DIC) (a, c, e, g, i, and k) and fluorescence (b, d, f, h, j, and l) images of *control(RNAi)* (a–d, and i–l) and *leo‐1(RNAi)* (e‐h) day 1 adult animals with *tjIs308[leo‐1p::GFP::leo‐1::leo‐1 3′‐UTR]* (a–h) or wild type (i–l). GFP::LEO‐1‐signals at the perinuclear region of the germ cells at the distal gonad were calculated (see Figure 1n). A rectangular area (8 μm × 40 μm) was used to analyze the GFP signals. c, d, g, h, k, and l are magnified images of the rectangular areas in a, b, e, f, i, and j, respectively. In all the panels, the anterior region of the gonad is to the left, and the dorsal region is at the top of the image. The posterior gonads are shown. The orange dotted lines mark the gonad boundaries. All fluorescent images were captured under identical exposure conditions. Scale bar (white), 50 μm.
**Figure S2** Analysis of the efficiency of RNAi knockdown of *pafo‐1*.
(a–l) Differential interference contrast (DIC) (a, c, e, g, i, and k) and fluorescence (b, d, f, h, j, and l) images of *control(RNAi)* (a–d and i–l) and *pafo‐1(RNAi)* (e–h) day 1 adult animals with *tjIs280[pafo‐1p::pafo‐1::mCherry::pafo‐1 3′‐UTR]* (a–h) or wild type (i–l). PAFO‐1::mCherry‐signals at the perinuclear region of the germ cells at the distal gonad were calculated (see Figure 1o). A rectangular area (8 μm × 40 μm) was chosen to analyze the mCherry signals. c, d, g, h, k, and l are magnified images of the rectangular areas in a, b, e, f, i, and j, respectively. In all the panels, the anterior region of the gonad is to the left, and the dorsal region is at the top of the image. The posterior gonads are shown. The orange dotted lines mark the gonad boundaries. All fluorescent images were captured under identical exposure conditions. Scale bar (white), 50 μm.

## Data Availability

All data and samples described in this work will be freely provided upon request.
