# Supplementary material for: The PAF1 complex cell autonomously promotes oogenesis in Caenorhabditis elegans
Source: Genes Cells. 2022 Apr 27;27(6):409–20. doi: 10.1111/gtc.12938 (PMC9321568; doi:10.1111/gtc.12938)
Supplement: Supplementary file 1 — Table S1 Caenorhabditis elegans strains constructed for this study. Table S2 Plasmids constructed for this study. [file GTC-27-409-s001.docx]

**SUPPLEMENTARY MATERIALS**

**FIGURE S1** Analysis of the efficiency of RNAi knockdown of *leo-1.*

(a–l) Differential interference contrast (DIC) (a, c, e, g, i, and k) and fluorescence (b, d, f, h, j, and l) images of *control(RNAi)* (a–d, and i–l) and *leo-1(RNAi)* (e-h) day 1 adult animals with *tjIs308[leo-1p::GFP::leo-1::leo-1 3′-UTR]* (a–h) or wild type (i–l). GFP::LEO-1-signals at the perinuclear region of the germ cells at the distal gonad were calculated (see Figure 1n). A rectangular area (8 μm × 40 μm) was used to analyze the GFP signals. c, d, g, h, k, and l are magnified images of the rectangular areas in a, b, e, f, i, and j, respectively. In all the panels, the anterior region of the gonad is to the left, and the dorsal region is at the top of the image. The posterior gonads are shown. The orange dotted lines mark the gonad boundaries. All fluorescent images were captured under identical exposure conditions. Scale bar (white), 50 μm.

**FIGURE S2** Analysis of the efficiency of RNAi knockdown of *pafo-1.*

(a–l) Differential interference contrast (DIC) (a, c, e, g, i, and k) and fluorescence (b, d, f, h, j, and l) images of *control(RNAi)* (a–d and i–l) and *pafo-1(RNAi)* (e–h) day 1 adult animals with *tjIs280[pafo-1p::pafo-1::mCherry::pafo-1 3′-UTR]* (a–h) or wild type (i–l). PAFO-1::mCherry-signals at the perinuclear region of the germ cells at the distal gonad were calculated (see Figure 1o). A rectangular area (8 μm × 40 μm) was chosen to analyze the mCherry signals. c, d, g, h, k, and l are magnified images of the rectangular areas in a, b, e, f, i, and j, respectively. In all the panels, the anterior region of the gonad is to the left, and the dorsal region is at the top of the image. The posterior gonads are shown. The orange dotted lines mark the gonad boundaries. All fluorescent images were captured under identical exposure conditions. Scale bar (white), 50 μm.

**Table S1** *Caenorhabditis elegans* strains constructed for this study.

| Strain name | Genotype |
| --- | --- |
| KUB20 | *leo-1(gk1081)/TmC5 IV[tmIs1220]* |
| KUB21 | *rtfo-1(tm5670)/TmC3 V[TmIs1230]* |
| KUB28 | *bkcSi11[oma-1p::oma-1::GFP::oma-1 3'-UTR, NeoR]* |
| KUB68 | *pafo-1(tm13347)/TmC3 V[TmIs1230]* |
| KUB77 | *bkcSi11[oma-1p::oma-1::GFP::oma-1 3'-UTR, NeoR] IV;pafo-1(tm13347)/TmC3 V[TmIs1230]* |
| KUB79 | *pafo-1(tm13347)/TmC 3V[TmIs1230];tjIs57[pie-1p::mCherry::H2B+unc-119(+)]; ozIs5 [gld-1::GFP + unc-119(+)]* |
| KUB87 | *pafo-1(tm13347)V; unc-119(ed3)Ⅲ?;tjIs280[pafo-1p::pafo-1::mCherry::pafo-1 3'-UTR, Cbr-unc-119(+)]* |
| KUB95 | *pafo-1(tm13347)V; unc-119(ed3)Ⅲ?;tjIs280[pafo-1p::pafo-1::mCherry, Cbr-unc-119(+)];bkcSi11 [oma-1p::oma-1::GFP::oma-1 3'-UTR, NeoR]* |
| KUB100 | *pafo-1(tm13347)V; bkcSi13[pie-1p::pafo-1::mCherry::pie-1 3'-UTR, NeoR]* |
| KUB107 | *pafo-1(tm13347)V; bkcSi12[pie-1p::pafo-1::mCherry::pie-1 3'-UTR, NeoR];bkcSi11 [oma-1p::oma-1::GFP::oma-1 3'-UTR, NeoR]* |
| KUB111 | *pafo-1(tm13347)V; bkcSi13[pie-1p::pafo-1::mCherry::pie-1 3'-UTR, NeoR];bkcSi11 [oma-1p::oma-1::GFP::oma-1 3'-UTR, NeoR]* |

**Table S2** Plasmids constructed for this study.

| Plasmid name | Transgene |
| --- | --- |
| pYK13 | *pCFJ910-MCS, NeoR miniMos vector with a new multi-cloning site* |
| pYK29 | *pYK13-oma-1p::oma-1(genome)::GFP::oma-1 3'-UTR* |
| pYK111 | *L4440_pafo-1* |
| pYK112 | *L4440_ctr-9 (1000bp)* |
| pYK113 | *L4440_leo-1* |
| pYK114 | *L4440_rtfo-1* |
| pYK114 | *L4440_cdc-73* |
| PYK232 | *pYK13-pie-1p::pafo-1(genome)::mCherry::pie-1 3'-UTR* |
