# Supplementary figures and images for: The PAF1 complex cell autonomously promotes oogenesis in Caenorhabditis elegans
Source: Genes Cells. 2022 Apr 27;27(6):409–20. doi: 10.1111/gtc.12938 (PMC9321568; doi:10.1111/gtc.12938)

DIC

fluorescent

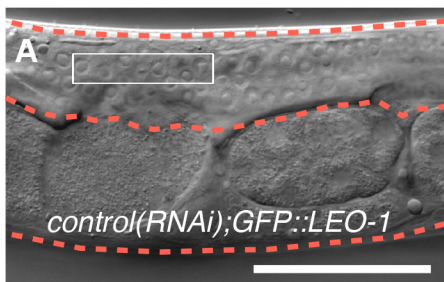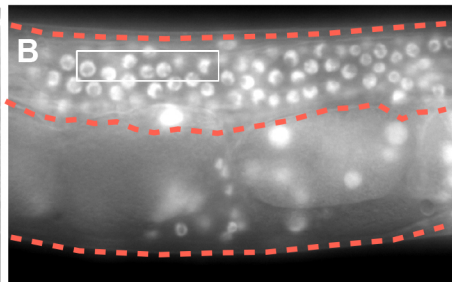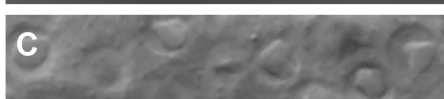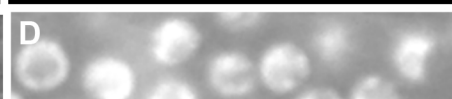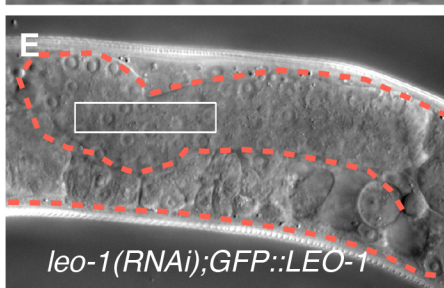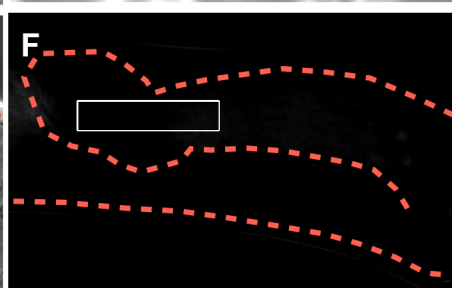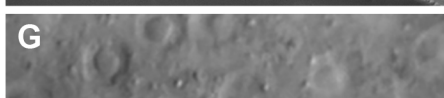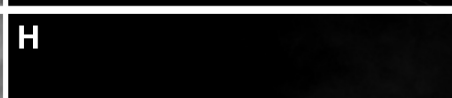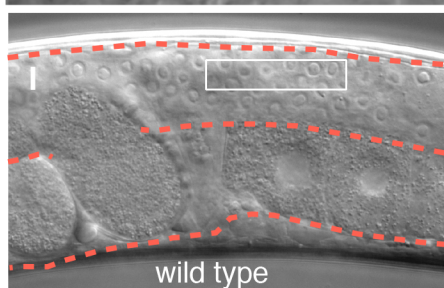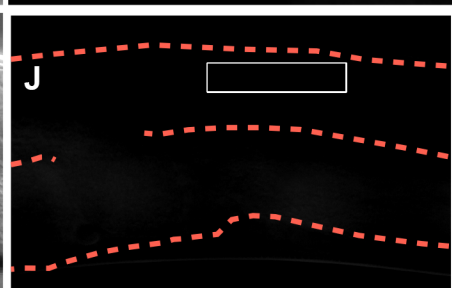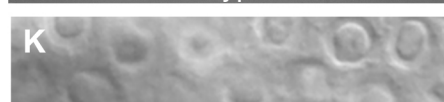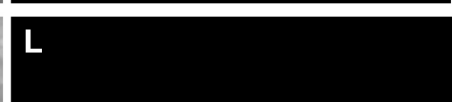

Figure S1

DIC

fluorescent

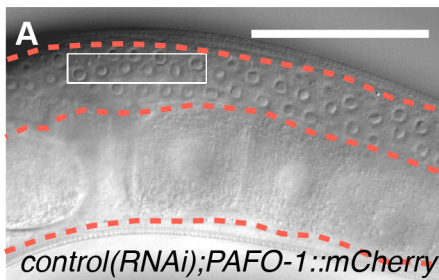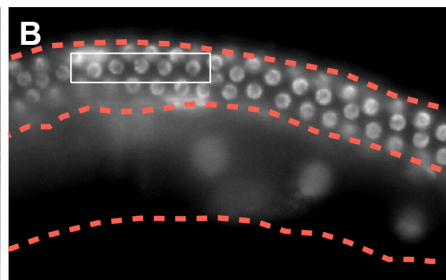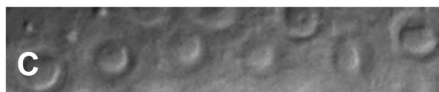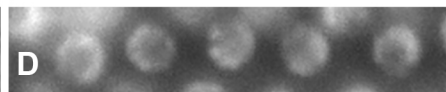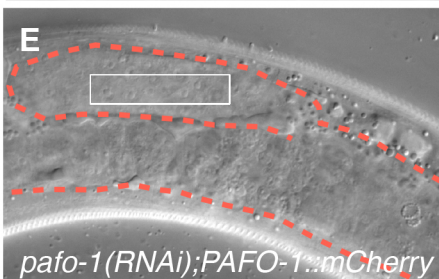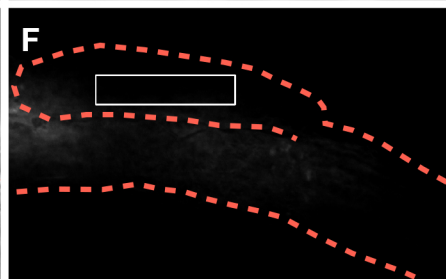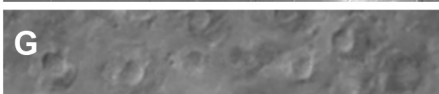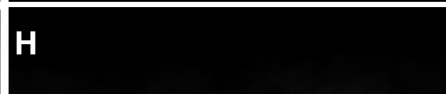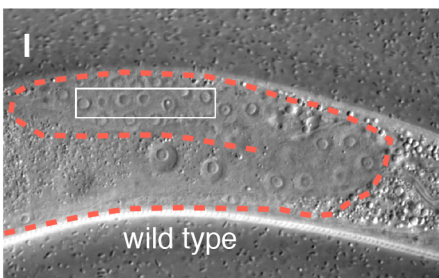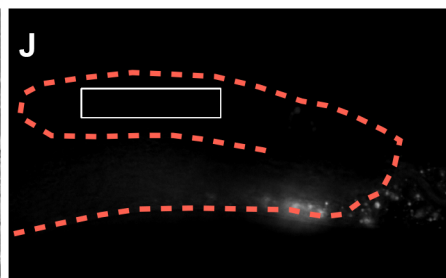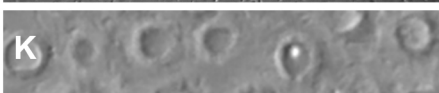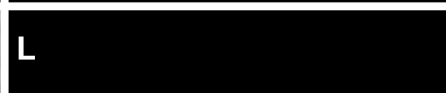

Figure S2

Supplement: Supplementary file 2 — FIGURE S1 Analysis of the efficiency of RNAi knockdown of leo‐1. (a–l) Differential interference contrast (DIC) (a, c, e, g, i, and k) and fluorescence (b, d, f, h, j, and l) images of control(RNAi) (a–d, and i–l) and leo‐1(RNAi) (e‐h) day 1 adult animals with tjIs308[leo‐1p::GFP::leo‐1::leo‐1 3′‐UTR] (a–h) or wild type (i–l). GFP::LEO‐1‐signals at the perinuclear region of the germ cells at the distal gonad were calculated (see Figure 1n). A rectangular area (8 μm × 40 μm) was used to analyze the GFP signals. c, d, g, h, k, and l are magnified images of the rectangular areas in a, b, e, f, i, and j, respectively. In all the panels, the anterior region of the gonad is to the left, and the dorsal region is at the top of the image. The posterior gonads are shown. The orange dotted lines mark the gonad boundaries. All fluorescent images were captured under identical exposure conditions. Scale bar (white), 50 μm. Figure S2 Analysis of the efficiency of RNAi knockdown of pafo‐1. (a–l) Differential interference contrast (DIC) (a, c, e, g, i, and k) and fluorescence (b, d, f, h, j, and l) images of control(RNAi) (a–d and i–l) and pafo‐1(RNAi) (e–h) day 1 adult animals with tjIs280[pafo‐1p::pafo‐1::mCherry::pafo‐1 3′‐UTR] (a–h) or wild type (i–l). PAFO‐1::mCherry‐signals at the perinuclear region of the germ cells at the distal gonad were calculated (see Figure 1o). A rectangular area (8 μm × 40 μm) was chosen to analyze the mCherry signals. c, d, g, h, k, and l are magnified images of the rectangular areas in a, b, e, f, i, and j, respectively. In all the panels, the anterior region of the gonad is to the left, and the dorsal region is at the top of the image. The posterior gonads are shown. The orange dotted lines mark the gonad boundaries. All fluorescent images were captured under identical exposure conditions. Scale bar (white), 50 μm. [file GTC-27-409-s002.pdf]
